# Interpretation of Confounding in Atomic Bomb Radiation Risk Studies

**DOI:** 10.2188/jea.JE20250454

**Published:** 2026-04-05

**Authors:** Kotaro Ozasa

**Affiliations:** Health Management Center, Kyoto Prefectural University of Medicine, Kyoto, Japan

**Keywords:** confounding, atomic bomb, radiation risk, the Life Span Study

Dear Editor,

It has been thought that, although no factors other than radiation exposure were used to evaluate atomic bomb radiation risks, the results have been accepted as scientifically reasonable.^1^ It is because survivors were non-selectively exposed to atomic bomb radiation, so that evaluation of radiation risk is mostly free from confounding, principally for analyses over the whole dose range. It was shown that there was almost no confounding due to smoking on radiation risk of cancer incidence^2^ or various factors on non-cancer mortality.^3^ However, those factors are rather important for risk evaluation for specific outcomes in different recently reported study schemes,^4^^,^^5^ as well as analysis at low dose levels. In those situations, biological and non-biological (mainly socioeconomic) causal associations are related complicatedly, so interpretation of confounding is confusing for investigation of biological effects of radiation exposure. Therefore, it is important to understand the relationship systematically, so that the following explanations with figure would be helpful.

Relevant factors are shown in Figure 1. First are the atomic bombing (A) and people’s exposure to atomic bomb radiation (B). The biologically caused health outcomes due to atomic bomb radiation (C) may occur directly or be mediated though some epidemiologically observable factors (D). Those factors may in turn be affected biologically and non-biologically by other factors (E and F). Some factors (E) were caused non-biologically from atomic bombing, typically socioeconomic damages caused by the bombing. Others (F) existed at the time of bombing but had non-causal association with the bombing or sometimes had reverse causal association (eg, residential location was consequently related to the distance from the hypocenter, which was definitely associated with individual radiation dose estimates).

**Figure 1.**
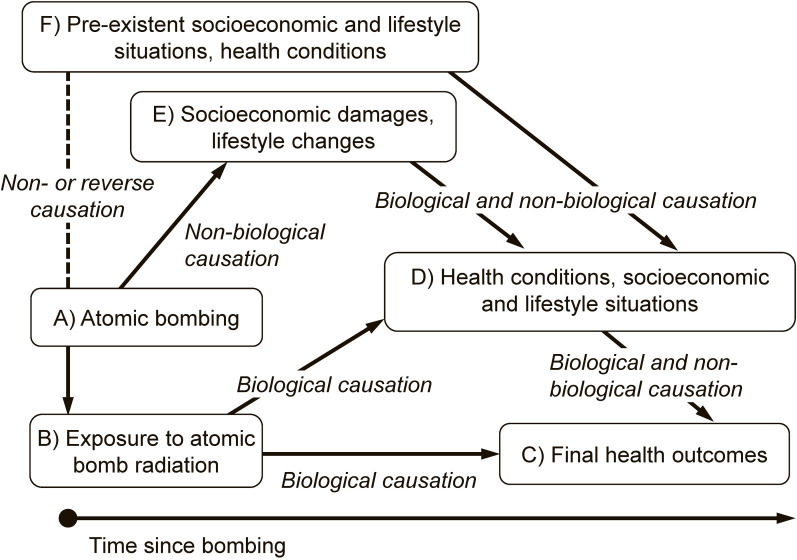
Association of relevant factors in understanding of confounding on radiation risk analysis among atomic bomb survivors and their children.

On evaluation of biological effects of exposure to atomic bomb radiation, non-biological causation should be treated as non-causal association. That is, all fractions of factors D coming from factors E and F should work as “confounding” for biological causation from B to C because factors E were caused non-biologically from the bombing and factors F already existed at the time of bombing. Fractions of factors D coming from exposure to atomic bomb radiation (B) should be included in the causal chain from B to C. In contrast, when all kinds of effects originating from the atomic bombing (A) are investigated, all pathways from A via B and E are included in the causal association, but “confounding” should be derived only from factors F.

Pre-existent factors (F) are not thought to be so serious for radiation risk analysis because those factors can be assumed to be randomized as survivors were non-selectively exposed to atomic bombing.^1^ The most possible exception is thought to be geospatial difference in socioeconomic situations of residents in Hiroshima and Nagasaki cities at the time of bombing. But, since distance from the hypocenter was related to the severity of socioeconomic damage due to bombing for survivors, it needs to be carefully considered whether observed socioeconomic status is pre-extent or caused by bombing.

When evaluating radiation risk at low dose levels, survivors distributed around outer wide areas and anticipated risk is small, so that those factors need to be carefully treated. In addition, the Life Span Study of atomic bomb survivors particularly includes people who were not in the cities of Hiroshima and Nagasaki at the time of bombing as a reference group. Characteristics and baseline health risk of these participants were different from in-city survivors.^6^ So, analysis applied adjustment for the not-in-city group.^2^

More complicated are the roles of factors D. In case that factors D are caused by both radiation exposure (B) and other factors (E, F), factors D work as both roles of “intermediate process in the biological causal association of radiation exposure” and “confounding.” Examples are shown in eFigure 1: atherosclerosis and hypertension as typical examples of both roles of factors D for ischemic heart disease and stroke^3^^,^^7^^,^^8^; eFigure 2: maternal and children’s conditions for health outcomes of children who were exposed to radiation in mother’s womb (*in utero*)^4^; eFigure 3: maternal conditions for congenital malformations and perinatal deaths of children who were conceived after parental exposure to radiation (filial-one generation)^5^ (in those two, both roles are thought to be important); and eFigure 4: follow-up of mortality in the filial-one generation, in which mostly no confounding is assumed.^9^ In actual analysis, it is usually difficult to quantify causal fractions from B and E to D, separately.

I hope that this letter fosters better understanding of epidemiological studies regarding atomic bomb radiation. Also, it could be applied to evaluation of radiation risk in situations with heavy social damage, like nuclear accidents. It is noted that responsibility for the contents of this letter lies solely with the current author, independent of referred articles’ authors, although they gave me important suggestions and I deeply appreciate them.

## SUPPLEMENTARY MATERIAL

eFigure 1. Ischemic heart disease and stroke.^3^^,^^7^^,^^8^ Atherosclerosis and hypertension (D) are thought to be caused by radiation exposure (B) and also influenced by other situations (E, F), so that they work as both roles of intermediate causation and confounding to the final outcomes of ischemic heart disease and stroke (C). In the Life Span Study of atomic bomb survivors, the role of confounding was thought be little. So, those factors need to be treated carefully as an intermediate cause. Factors D with asterisk^*^ are thought to be caused biologically from radiation exposure (B).

eFigure 2. Health outcomes of children who were exposed to radiation in mother’s womb (*in utero*).^4^ Small head size and low birth weight of the child (D) are thought to be directly caused by intrauterine exposure to radiation (B) and, in addition, influenced by reduced maternal ability of keeping pregnancy due to maternal radiation exposure (D) that was derived directly by radiation exposure (B) and indirectly via socioeconomical damages due to bombing (E). Parental socioeconomic damage during pregnancy and after birth of the child (D) could be derived from bombing via factors E. Pre-extent biological dispositions and other situations (F) could also affect those conditions (D). Therefore, the final health outcomes (C) are thought to be caused by direct biological effects of intrauterine radiation exposure (B to C) and indirect biological effects of radiation exposure (B to C via E) and also confounded by non-biological causation of atomic bombing (B to C via E) and pre-existent factors (F). Probably, the three pathways except for the last (F) would be important for the final health outcomes. So, the results unadjusted for factor D would include confounding due to the third pathway while adjustment for factor D would over-adjust the second pathway of indirect causations. Factors D with asterisk^*^ are thought to be caused biologically from radiation exposure (B).

eFigure 3. Congenital malformations and perinatal deaths of survivors’ children conceived after parental exposure to radiation (filial-one generation).^5^ Congenital malformations and perinatal deaths (C) are thought to be not only caused by genetic effects of radiation exposure on parental germ cells (B) but possibly affected by damaged intrauterine circumstances and reduced maternal ability of keeping pregnancy that were caused directly by maternal radiation exposure (B). Those damages were also influenced by socioeconomic damage due to bombing (E), especially because this study was conducted shortly after the war, and pre-existent biological dispositions and other situations (F). Therefore, the final health outcomes (C) are thought to be caused by direct biological effects of radiation exposure on parental germ cells (B to C) and indirect biological effects of radiation exposure on mother (B to C via E) and also confounded by non-biological causation of atomic bombing (B to C via E) and pre-existent factors (F). The three pathways except for the last (F) would be important for the final health outcomes, in this study, too. But, different from the above (2), no parameters for factors D were available. Factors D with asterisk^*^ are thought to be caused biologically from radiation exposure (B).

eFigure 4. Follow-up of mortality in the filial-one generation.^9^ Relevant factors were similar to the above study (3) as this study purposed to evaluate genetic effects of parental exposure to radiation on long-term cancer and non-cancer mortality. In the paper, it was noted that children conceived shortly after the bombing had higher risk of non-cancer disease death and those deaths were not thought to derive from radiation exposure, but were likely related to bad hygiene and socioeconomic situations shortly after the war,^9^ which indicated the role of confounding by factors D in this figure. For other children who were born later, the impact of factors D were small as such situations were recovered along with the period after the war. So, it is thought that the impact of factors D was generally small on the observed results for a long period since exposure to radiation. Factors D with asterisk^*^ are thought to be caused biologically from radiation exposure (B).
