# Supplementary material for: Interpretation of Confounding in Atomic Bomb Radiation Risk Studies
Source: J Epidemiol. 2026 Apr 5;36(4):153–4. doi: 10.2188/jea.JE20250454 (PMC12975772; doi:10.2188/jea.JE20250454)
Supplement: Supplementary file 1 [file je-36-153-s001.pdf]

**eFigure 1.** Ischemic heart disease and stroke

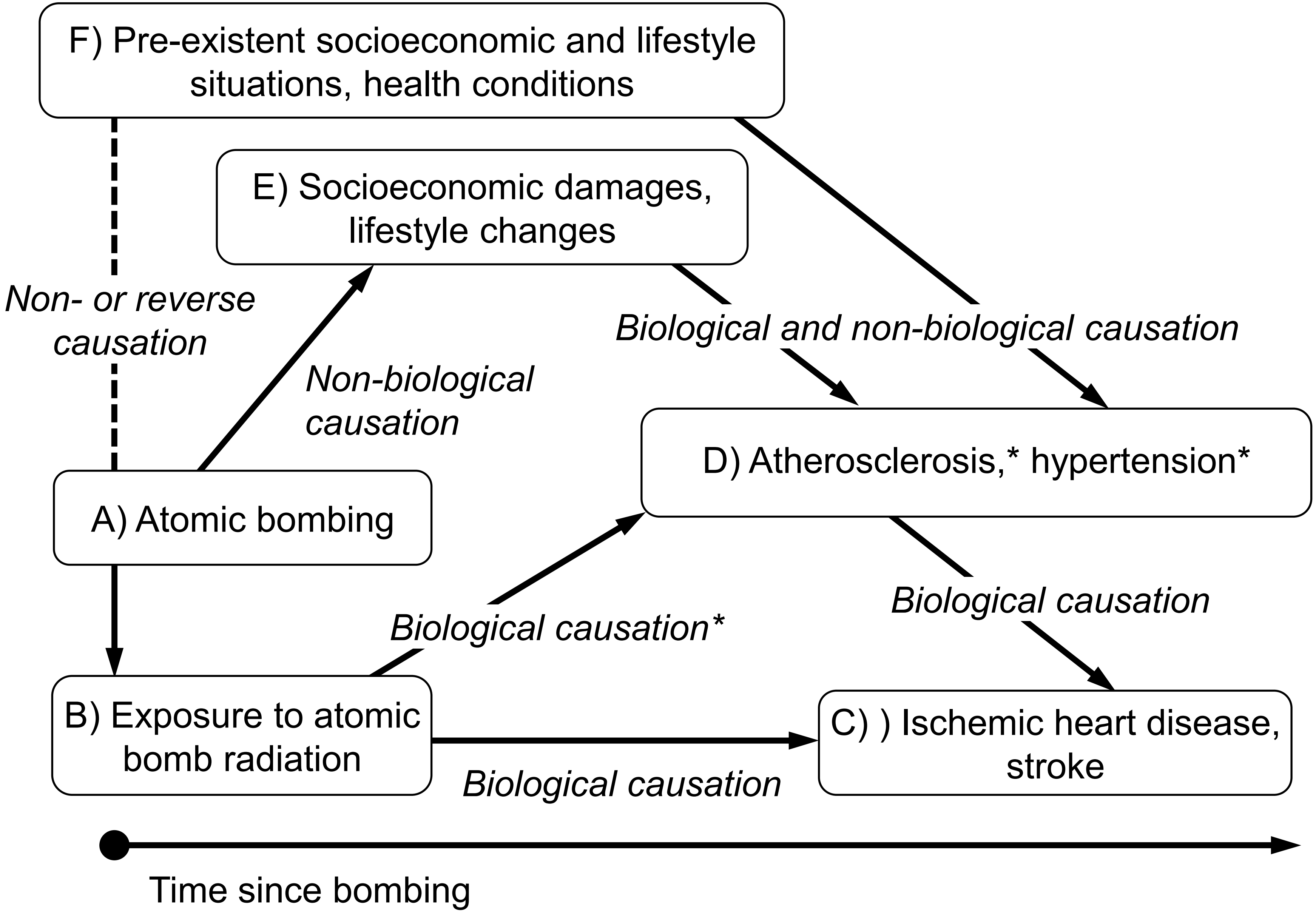

**eFigure 2.** Health outcomes of children who were exposed to radiation in mother's womb (*in utero*)

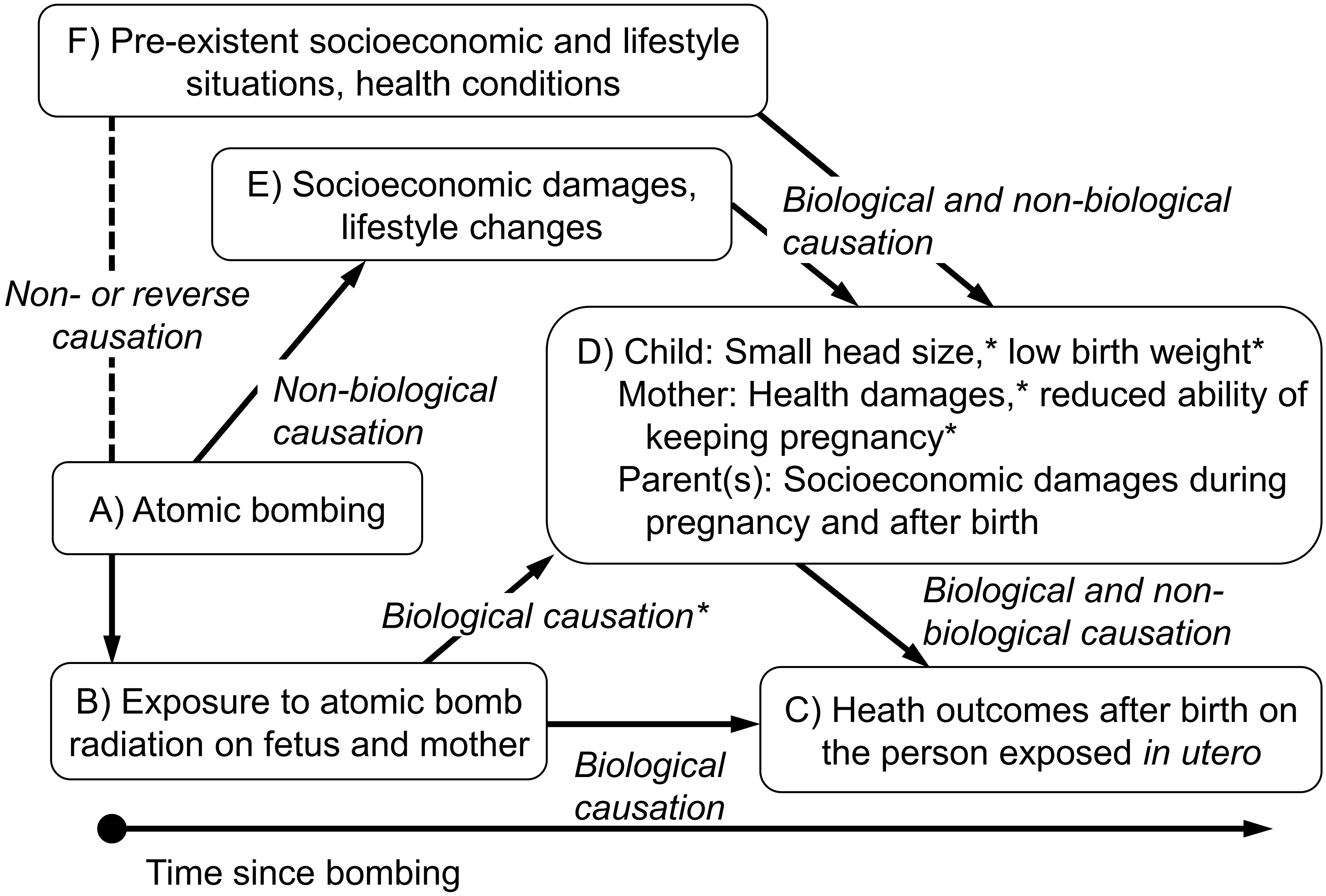

**eFigure 3.** Congenital malformations and perinatal deaths of survivors' children conceived after parental exposure to radiation (filial-one generation)

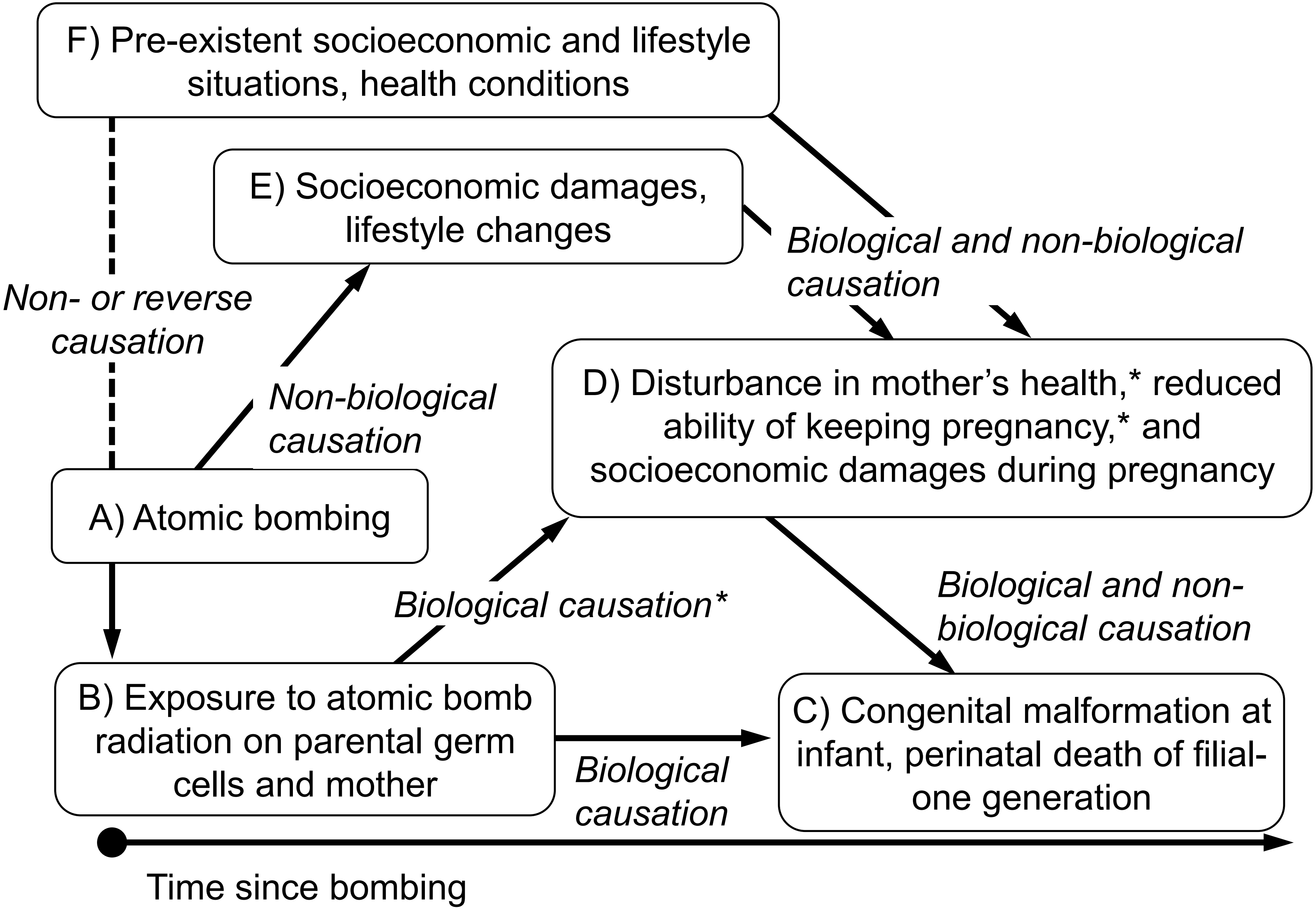

**eFigure 4.** Follow-up of mortality in the filial-one generation

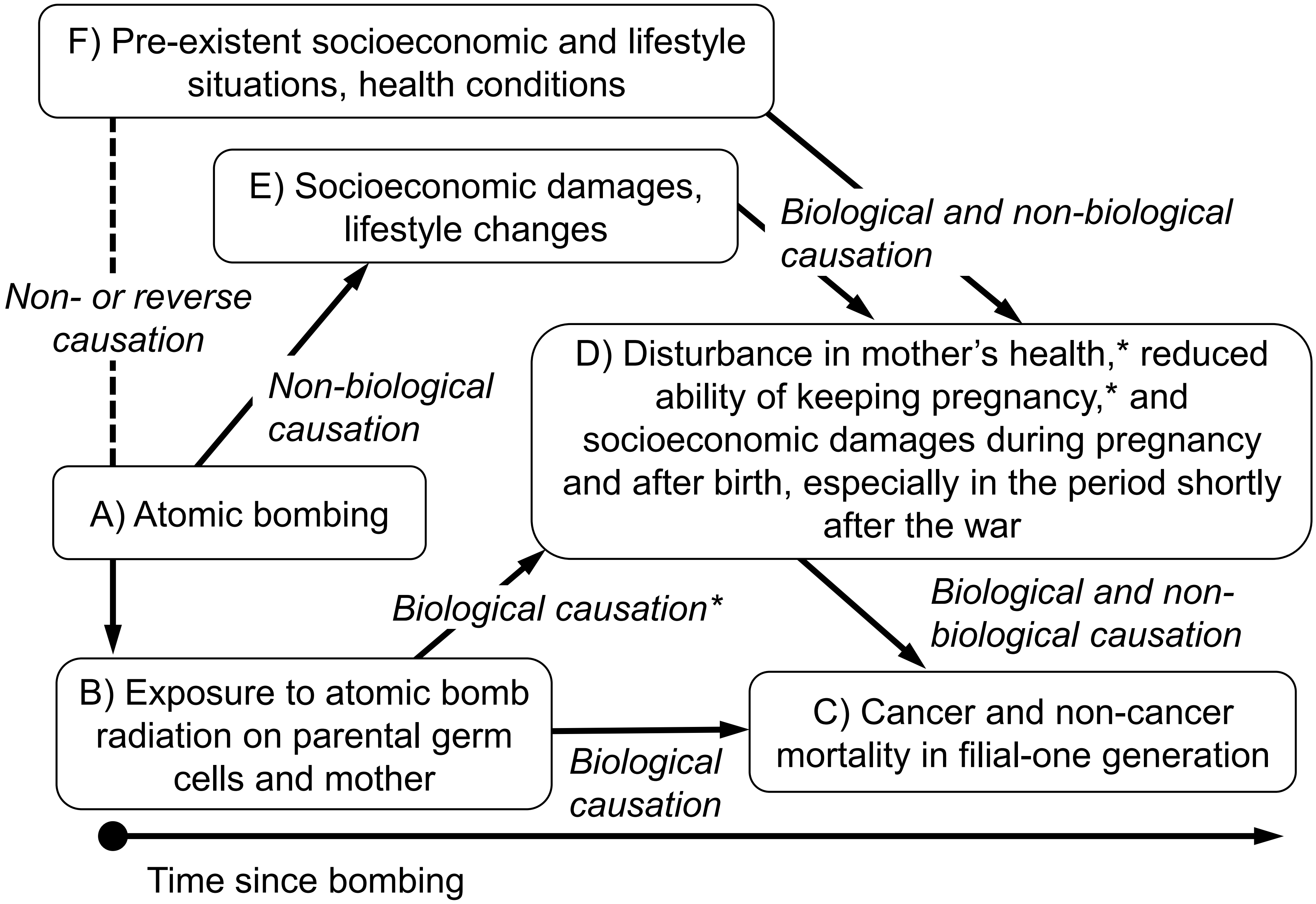

**eFigure Legend**

**eFigure 1.** Ischemic heart disease and stroke.[3, 7, 8] Atherosclerosis and hypertension (D) are thought to be caused by radiation exposure (B) and also influenced by other situations (E, F), so that they work as both roles of intermediate causation and confounding to the final outcomes of ischemic heart disease and stroke (C). In the Life Span Study of atomic bomb survivors, the role of confounding was thought be little. So, those factors need to be treated carefully as an intermediate cause. Factors D with asterisk* are thought to be caused biologically from radiation exposure (B).

**eFigure 2.** Health outcomes of children who were exposed to radiation in mother's womb (*in utero*).[4] Small head size and low birth weight of the child (D) are thought to be directly caused by intrauterine exposure to radiation (B) and, in addition, influenced by reduced maternal ability of keeping pregnancy due to maternal radiation exposure (D) that was derived directly by radiation exposure (B) and indirectly via socioeconomical damages due to bombing (E). Parental socioeconomic damage during pregnancy and after birth of the child (D) could be derived from bombing via factors E. Pre-extent biological dispositions and other situations (F) could also affect those conditions (D). Therefore, the final health outcomes (C) are thought to be caused by direct biological effects of intrauterine radiation exposure (B to C) and indirect biological effects of radiation exposure (B to C via E) and also confounded by non-biological causation of atomic bombing (B to C via E) and pre-existent factors (F). Probably, the three pathways except for the last (F) would be important for the final health outcomes. So, the results unadjusted for factor D would include confounding due to the third pathway while adjustment for factor D would over-adjust the second pathway of indirect causations. Factors D with asterisk* are thought to be caused biologically from radiation exposure (B).

**eFigure 3.** Congenital malformations and perinatal deaths of survivors' children conceived after parental exposure to radiation (filial-one generation).[5] Congenital malformations and perinatal deaths (C) are thought to be not only caused by genetic effects of radiation exposure on parental germ cells (B) but possibly affected by damaged intrauterine circumstances and reduced maternal ability of keeping pregnancy that were caused directly by maternal radiation exposure (B). Those damages were also influenced by socioeconomic damage due to bombing (E), especially because this study was conducted shortly after the war, and pre-existent biological dispositions and other situations (F). Therefore, the final health outcomes (C) are thought to be caused by direct biological effects of radiation exposure on parental germ cells (B to C) and indirect biological effects of radiation exposure on mother (B to C via E) and also confounded by non-biological causation of atomic bombing (B to C via E) and pre-existent factors (F). The three pathways except for the last (F) would be important for the final health outcomes, in this study, too. But, different from the above (2), no parameters for factors D were available. Factors D with asterisk* are thought to be caused biologically from radiation exposure (B).

**eFigure 4.** Follow-up of mortality in the filial-one generation.[9] Relevant factors were similar to the above study (3) as this study purposed to evaluate genetic effects of parental exposure to radiation on long-term cancer and non-cancer mortality. In the paper, it was noted that children conceived shortly after the bombing had higher risk of non-cancer disease death and those deaths were not

thought to derive from radiation exposure, but were likely related to bad hygiene and socioeconomic situations shortly after the war,[9] which indicated the role of confounding by factors D in this figure. For other children who were born later, the impact of factors D were small as such situations were recovered along with the period after the war. So, it is thought that the impact of factors D was generally small on the observed results for a long period since exposure to radiation. Factors D with asterisk* are thought to be caused biologically from radiation exposure (B).
